# Risk Factors Associated With Ischemic Stroke in Japanese Patients With Nonvalvular Atrial Fibrillation

**DOI:** 10.1001/jamanetworkopen.2020.2881

**Published:** 2020-04-15

**Authors:** Ken Okumura, Hirofumi Tomita, Michikazu Nakai, Eitaro Kodani, Masaharu Akao, Shinya Suzuki, Kenshi Hayashi, Mitsuaki Sawano, Masahiko Goya, Takeshi Yamashita, Keiichi Fukuda, Hisashi Ogawa, Toyonobu Tsuda, Mitsuaki Isobe, Kazunori Toyoda, Yoshihiro Miyamoto, Hiroaki Miyata, Tomonori Okamura, Yusuke Sasahara

**Affiliations:** 1 Department of Cardiology, Hirosaki University Graduate School of Medicine, Hirosaki, Japan; 2 Division of Cardiology, Saiseikai Kumamoto Hospital, Kumamoto, Japan; 3 Center for Cerebral and Cardiovascular Disease Information, National Cerebral and Cardiovascular Center, Suita, Japan; 4 Department of Internal Medicine and Cardiology, Nippon Medical School Tama-Nagayama Hospital, Tokyo, Japan; 5 Department of Cardiology, National Hospital Organization Kyoto Medical Center, Kyoto, Japan; 6 Department of Cardiovascular Medicine, The Cardiovascular Institute, Tokyo, Japan; 7 Department of Cardiovascular Medicine, Kanazawa University Graduate School of Medical Science, Kanazawa, Japan; 8 Department of Cardiology, Keio University School of Medicine, Tokyo, Japan; 9 Department of Cardiovascular Medicine, Tokyo Medical and Dental University, Tokyo, Japan; 10 Sakakibara Heart Institute, Tokyo, Japan; 11 Department of Cerebrovascular Medicine, National Cerebral and Cardiovascular Center, Suita, Japan; 12 Department of Preventive Cardiology, National Cerebral and Cardiovascular Center, Suita, Japan; 13 Department of Health Policy and Management School of Medicine, Keio University, Tokyo, Japan; 14 Department of Preventive Medicine and Public Health, Keio University School of Medicine, Tokyo, Japan

## Abstract

**Importance:**

Despite the development of risk stratification schemes that have been widely used to determine the risk for thromboembolism in patients with nonvalvular atrial fibrillation (NVAF), risk stratification schemes in Asian patients with NVAF remain undetermined.

**Objective:**

To determine risk factors for ischemic stroke in Japanese patients with NVAF.

**Design, Setting, and Participants:**

This cohort study analyzed individual patient data from 5 AF registries in Japan: J-RHYTHM (Japanese Rhythm Management Trial for Atrial Fibrillation) Registry, Fushimi AF Registry, Shinken Database, Keio Interhospital Cardiovascular Studies (Keio Study), and Hokuriku-Plus AF Registry. Patients with atrial fibrillation were registered from 158 institutions in the J-RHYTHM Registry, 80 in the Fushimi AF Registry, a single hospital in Shinken Database, 11 in the Keio Study, and 19 in the Hokuriku-Plus AF Registry. Patients with valvular AF or lacking data were excluded. Data were collected and integrated in March 2016, and those from the Keio Study were updated in April 2018. Data were analyzed from April 2018 to February 2020.

**Main Outcomes and Measures:**

Significant risk factors for ischemic stroke were determined by adjusted Cox proportional hazards analysis.

**Results:**

In total, 12 289 patients with NVAF (3758 [31%] female; mean [SD] age 70.2 [11] years) were analyzed with a mean (SD) follow-up period of 649 (181) days (1.8 [0.5] years). During 21 820 person-years of follow-up, 241 cases of ischemic stroke were reported. Risk factors associated with ischemic stroke after adjustment for oral anticoagulant use at enrollment were age (75-84 years: hazard ratio [HR], 1.74; 95% CI, 1.32-2.30; *P* < .001; and ≥85 years: HR, 2.41; 1.63-3.56; *P* < .001), hypertension (HR, 1.60; 95% CI, 1.15-2.23; *P* = .006), previous stroke (HR, 2.75; 95% CI, 2.09-3.62; *P* < .001), persistent or permanent AF (HR, 1.59; 95% CI, 1.21-2.10; *P* = .001), and body mass index less than 18.5 (HR, 1.55; 95% CI, 1.05-2.29; *P* = .03). Neither diabetes nor heart failure were identified as risk factors for ischemic stroke.

**Conclusions and Relevance:**

Previous stroke, advanced age, hypertension, persistent or permanent AF, and low body mass index were independent risk factors associated with ischemic stroke in Japanese patients with NVAF.

## Key Points

**Question:**

What risk factors are associated with ischemic stroke in Japanese patients with nonvalvular atrial fibrillation?

**Findings:**

This cohort study examined data from 12 289 individual patients in 5 atrial fibrillation registries in Japan and found that previous stroke, advanced age, hypertension, type of atrial fibrillation, and low body mass index were significant, independent risk factors associated with ischemic stroke.

**Meaning:**

These risk factors may be useful for identifying patients with nonvalvular atrial fibrillation who are at risk for ischemic stroke.

## Teaser

This cohort study pools data from several registries of Japanese patients with nonvalvular atrial fibrillation (NVAF) to assess various risk factors for stroke associated with NVAF.

## Introduction

The CHA_2_DS_2_-VASc score (congestive heart failure, hypertension, age 75 years or older, diabetes, prior stroke/transient ischemic attack–vascular disease, age 65-74 years, sex category) is widely used in Europe and the United States for thromboembolic risk stratification in patients with nonvalvular atrial fibrillation (NVAF),^1,2^ whereas CHADS_2_ (congestive heart failure, hypertension, aged 75 years or older, diabetes, prior stroke/transient ischemic attack) score is used in Japan.^3^ In a previous study,^4^ we showed that both scores are useful for risk stratification in Japanese NVAF patients. But all CHADS_2_ score factors may not necessarily represent risks for thromboembolism. Our previous pooled data of the J-RHYTHM (Japanese Rhythm Management Trial for Atrial Fibrillation) Registry, Fushimi AF Registry, and Shinken Database showed that, for patients without oral anticoagulants (OAC), age 75 years or older, hypertension, and previous stroke or transient ischemic attack were independent risk factors associated with ischemic stroke, but diabetes or heart failure were not.^5^ The pooled data also showed that the annual ischemic stroke rate was 0.9%, 1.5%, and 2.7% in individuals with a CHADS_2_ score of 1, 2, and 3, respectively, which was lower than those shown in the original data.^5,6^ These findings suggest some differences in risk factors associated with thromboembolism by race and region.

Several novel risk factors in Japanese patients have been identified in the Fushimi AF, J-RHYTHM, and Hokuriku-Plus AF registries, including persistent or permanent AF, low body weight, anemia, and kidney dysfunction.^7,8,9,10,11^ Furthermore, female sex was shown not to be a risk for thromboembolism in the Japanese population.^12^ However, these results were obtained from a Japanese registry in which the patient demographic characteristics, especially mean age and risk for thromboembolism, were somewhat different from each other. In the present study, we pooled the data from the 5 AF prospective registries in Japan and assessed risk factors associated with ischemic stroke in the Japanese patients with NVAF.

## Methods

### Study Patients

We pooled the data from 5 major AF prospective registries in Japan: J-RHYTHM Registry (7937 patients),^13^ Fushimi AF Registry (3749 patients),^14^ Shinken Database (2957 patients),^15^ Keio Interhospital Cardiovascular Studies (Keio Study) (783 patients),^16^ and Hokuriku-Plus AF Registry (1492 patients).^11^ The data from each registry were collected and integrated in March 2016, and those from the Keio Study were updated in April 2018. A total of 819 patients with valvular AF and 3810 lacking complete data were excluded (Figure; eTable 1 in the Supplement). In total, 12 289 patients with NVAF were analyzed. Among the data collected in each registry, age, sex, congestive heart failure, hypertension, diabetes, previous stroke, vascular disease, type of AF (paroxysmal vs persistent or permanent), body mass index (BMI), creatinine level, and administration of OAC at enrollment were analyzed in this study. Researchers determined CHADS_2_ and CHA_2_DS_2_-VASc scores for each patient at enrollment. In this study, we focused on the incidence of ischemic stroke events. To balance the follow-up period among the registries, event data from individuals whose follow-up period exceeded 730 days were excluded from the analysis.

**Figure.**
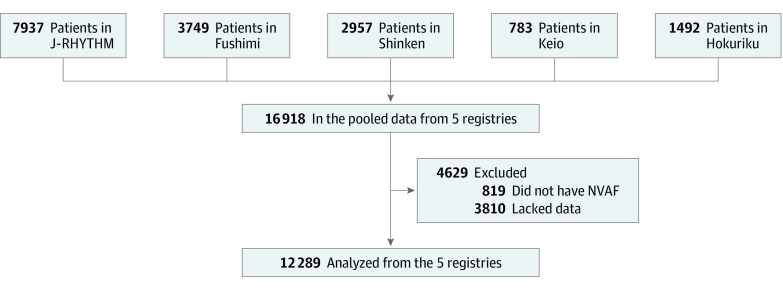
Flowchart of the Study Patients^a^ ^a^Registries included in this study are: J-RHYTHM (Japanese Rhythm Management Trial for Atrial Fibrillation) Registry, Fushimi AF Registry, Shinken Database, Keio Interhospital Cardiovascular Studies, and Hokuriku-Plus AF Registry. NVAF indicates nonvalvular atrial fibrillation.

This study was approved and an exemption of informed consent was granted by the ethics committee of the Hirosaki University Graduate School of Medicine, Hirosaki, Japan, because the integrated data used did not include personally identifiable information. This study followed Strengthening the Reporting of Observational Studies in Epidemiology (STROBE) reporting guidelines.

### Characteristics of Each Registry

The patient demographic characteristics of each registry have been published previously.^11^^,^^13,14,15,16^ Briefly, the J-RHYTHM Registry was a prospective, observational cohort study aiming to determine the current status of anticoagulation therapy in Japanese patients with AF.^13^ A total of 7937 patients with AF, with or without warfarin treatment, from 158 institutions throughout Japan were registered between January and July 2009. The Fushimi AF Registry was a community-based survey of patients with AF aiming to enroll all patients in Fushimi-ku, Kyoto, Japan.^14^ It enrolled 3183 patients with or without OAC. The Shinken Database was a prospective cohort study enrolling new patients with AF who visited the Cardiovascular Institute Hospital in Tokyo, Japan.^15^ The Keio Study was a multicenter registry-based retrospective cohort study collecting outcome data from consecutive newly diagnosed patients with AF at 11 hospitals within the Kanto area of Japan.^16^ The Hokuriku-Plus AF Registry was a prospective, observational cohort study enrolling patients from 19 institutions in the Hokuriku and Yokohama areas of Japan.^11^ Patient registration period and definition of risk factors (congestive heart failure, hypertension, diabetes, vascular diseases) in each registry are shown in eTable 2 in the Supplement.

### Statistical Analysis

Data were expressed as mean (standard deviation) or number (percentage). Kaplan-Meier analysis was conducted with numbers at risk shown in 0-, 200-, 400-, and 600-day intervals. Cox proportional hazards models were used to calculate hazard ratio (HR) and 95% confidence interval for incidence of ischemic stroke events; significant risk factors were estimated. Also, a competing risk regression model was used to consider death prior to stroke. To account for missing data, multiple imputation analysis with 20 imputations was performed. Furthermore, shared frailty analysis was performed to assess differences in the registries. Statistical analyses were performed using STATA version 16 (StataCorp). Statistical significance was set at 2-sided *P* < .05.

## Results

### Clinical Profiles of the Study Patients

Table 1 shows the profiles of the 12 289 patients with NVAF (mean [SD] age, 70.2 [11.0] years; 3758 [31%] female). Mean (SD) CHADS_2_ and CHA_2_DS_2_-VASc scores were 1.7 (1.3) and 2.9 (1.7), respectively. The numbers of patients with no OAC, warfarin, and direct oral anticoagulant (DOAC) were 3197 (26%), 7886 (64%), and 1206 (10%), respectively. Mean (SD) follow-up was 649 (181) days (1.8 [0.5] years).

**Table 1.** Characteristics of the Study Patients^a^

Characteristic	No. (%)					
	Total (N = 12 289)	J-RHYTHM (n = 5928)	Fushimi (n = 2971)	Shinken (n = 1534)	Keio (n = 553)	Hokuriku (n = 1303)
Age, y						
Mean (SD)	70.2 (11)	69.7 (10)	74.1 (11)	63.7 (12)	66.5 (12)	72.4 (10)
<75	7665 (63)	3897 (66)	1398 (47)	1246 (81)	405 (73)	719 (55)
75-84	3735 (30)	1773 (30)	1130 (38)	251 (16)	122 (22)	459 (35)
≥85	889 (7)	258 (4)	443 (15)	37 (3)	26 (5)	125 (10)
Female	3758 (31)	1707 (29)	1182 (40)	361 (24)	152 (28)	356 (27)
Congestive heart failure	3258 (27)	1698 (29)	876 (30)	180 (12)	85 (15)	419 (32)
Hypertension	8971 (73)	4608 (78)	2344 (79)	660 (43)	469 (85)	890 (68)
Diabetes	2541 (21)	1119 (19)	746 (25)	223 (15)	88 (16)	365 (28)
Previous stroke	1729 (14)	843 (14)	582 (20)	79 (5)	47 (9)	178 (14)
Vascular disease	1703 (14)	661 (11)	566 (19)	118 (8)	69 (13)	289 (22)
CHADS_2_ score, mean (SD)	1.7 (1.3)	1.7 (1.2)	2.1 (1.3)	1.2 (1.1)	1.3 (1.2)	2.0 (1.3)
0	1965 (16)	893 (15)	287 (10)	469 (30)	157 (29)	159 (12)
1	3920 (32)	2041 (34)	794 (26)	533 (35)	190 (34)	362 (28)
≥2	6404 (52)	2994 (51)	1890 (64)	532 (35)	206 (37)	782 (60)
CHA_2_DS_2_-VASc score, mean (SD)	2.9 (1.7)	2.8 (1.6)	3.5 (1.7)	2.0 (1.6)	2.3 (1.8)	3.3 (1.7)
0	890 (7)	372 (6)	89 (3)	297 (19)	85 (15)	47 (4)
1	1830 (15)	901 (15)	290 (10)	370 (24)	121 (22)	148 (11)
≥2	9569 (78)	4655 (79)	2592 (87)	867 (57)	337 (63)	1108 (85)
Type of atrial fibrillation						
Paroxysmal	5504 (45)	2254 (38)	1456 (49)	979 (64)	319 (58)	496 (38)
Persistent or permanent	6785 (55)	3674 (62)	1515 (51)	555 (36)	234 (42)	807 (62)
Body mass index^b^						
<18.5	895 (7)	363 (6)	336 (11)	84 (6)	33 (6)	79 (6)
18.5-24.9	7562 (62)	3685 (62)	1811 (61)	930 (61)	339 (61)	797 (61)
≥25	3832 (31)	1880 (32)	824 (28)	520 (34)	181 (33)	427 (33)
Creatinine ≥1.0 mg/dL	3821 (31)	1927 (32)	951 (32)	398 (26)	168 (30)	377(29)
Low hemoglobin^c^	3256 (27)	1373 (23)	1137 (38)	303 (20)	89 (16)	354 (27)
Follow-up period, mean (SD), d	649 (181)	682 (136)	612 (197)	547 (273)	711 (87)	672 (152)
Oral anticoagulant						
None	3197 (26)	745 (13)	1373 (46)	783 (51)	107 (19)	189 (15)
Warfarin	7886 (64)	5183 (87)	1361 (46)	528 (34)	119 (22)	695 (53)
Direct oral anticoagulant	1206 (10)	0	237 (8)	223 (15)	327 (59)	419 (32)

Abbreviations: AF, atrial fibrillation; CHADS_2_, congestive heart failure, hypertension, age 75 years or older, diabetes, prior stroke/transient ischemic attack; CHA_2_DS_2_-VASc, congestive heart failure, hypertension, age 75 years or older, diabetes, prior stroke/transient ischemic attack–vascular disease, age 65-74, sex category.

SI conversion factors: To convert creatinine to μmol/L, multiply by 76.25; hemoglobin to g/L, multiply by 10.0.

^a^ Individual patient data from 5 atrial fibrillation registries in Japan: Japanese Rhythm Management Trial for Atrial Fibrillation Registry (J-RHYTHM), Fushimi AF Registry (Fushimi), Shinken Database (Shinken), Keio Interhospital Cardiovascular Studies (Keio), and Hokuriku-Plus AF Registry (Hokuriku) were collected and integrated.

^b^ Calculated as weight in kilograms divided by height in meters squared.

^c^ Low hemoglobin was defined as less than 13 g/dL for male participants and less than 12 g/dL for female participants.

### Risk Factors Associated With Ischemic Stroke

During 21 820 person-years of follow-up, 241 cases of ischemic stroke were reported. Univariate, multivariable, and stepwise Cox proportional hazards models were used to determine significant risk factors for the incidence of ischemic stroke (Table 2). A stepwise analysis showed that age (75-84 years: HR, 1.74; 95% CI, 1.32-2.30; *P* < .001; and ≥85 years: HR, 2.41; 95% CI, 1.63-3.56; *P* < .001), hypertension (HR, 1.60; 95% CI, 1.15-2.23; *P* = .006), previous stroke (HR, 2.75; 95% CI, 2.09-3.62; *P* < .001), persistent or permanent AF (HR, 1.59; 95% CI, 1.21-2.10; *P* = .001), and BMI less than 18.5 (calculated as weight in kilograms divided by height in meters squared) (HR, 1.55; 95% CI, 1.05-2.29; *P* = .03) were significant risk factors associated with ischemic stroke after adjusting OAC administration at enrollment. Multivariable analysis also supported these results (Table 2). Of note, 57 patients whose only risk factor was low BMI had no ischemic stroke event.

**Table 2.** Univariate and Multivariable Analyses by Cox Proportional Hazards Model

Characteristic	Univariate		Multivariable		Stepwise	
	HR (95% CI)	*P* value	HR (95% CI)	*P* value	HR (95% CI)	*P* value
Age, y						
<75	1 [Reference]		1 [Reference]		1 [Reference]	
75-84	2.03 (1.54,2.67)	<.001	1.60 (1.19-2.13)	.002	1.74 (1.32-2.30)	<.001
≥85	3.57 (2.46-5.18)	<.001	2.04 (1.34-3.09)	.001	2.41 (1.63-3.56)	<.001
Female	1.36 (1.05-1.76)	.02	1.19 (0.90-1.58)	.22		
Congestive heart failure	1.48 (1.14-1.93)	.004	1.08 (0.81-1.44)	.59		
Hypertension	1.63 (1.18-2.27)	.003	1.51 (1.08-2.12)	.02	1.60 (1.15-2.23)	.006
Diabetes	1.21 (0.90-1.62)	.21	1.09 (0.81-1.47)	.57		
Previous stroke	3.08 (2.36-4.03)	<.001	2.72 (2.06-3.58)	<.001	2.75 (2.09-3.62)	<.001
Vascular disease	1.34 (0.96-1.87)	.09	1.03 (0.73-1.46)	.85		
Persistent or permanent atrial fibrillation	1.61 (1.23-2.11)	<.001	1.58 (1.19-2.10)	.002	1.59 (1.21-2.10)	.001
Body mass index <18.5^a^	1.96 (1.34-2.87)	.001	1.47 (0.98-2.19)	.06	1.55 (1.05-2.29)	.03
Creatinine ≥1.0 mg/dL	1.47 (1.13-1.90)	.004	1.21 (0.92-1.60)	.18		
Low hemoglobin^b^	1.73 (1.33-2.24)	<.001	1.18 (0.88-1.57)	.26		
No oral anticoagulant	1.49 (1.14-1.95)	.004	1.86 (1.40-2.47)	<.001	1.86 (1.40-2.47)	<.001

SI conversion factors: To convert creatinine to μmol/L, multiply by 76.25; hemoglobin to g/L, multiply by 10.0.

^a^ Calculated as weight in kilograms divided by height in meters squared.

^b^ Low hemoglobin is defined as less than 13 g/dL for male participants and less than 12 g/dL for female participants.

We observed 26 deaths prior to the incidence of an ischemic stroke event. We evaluated the effect of these deaths on the data using a competing risk regression model, and the analysis showed similar results (eTable 3 in the Supplement). The event rate in the missing population (0.82 per 100 person-years) was similar or a bit lower compared with that in the study population (1.15 per 100 person-years), while the missing population had a lower mean (SD) age (67.6 [12] years) and slightly lower CHADS_2_ score (1.4 [1.2]) than the study population. Furthermore, we performed multiple imputation analysis with 20 imputations and shared frailty analysis, which showed similar results (data not shown). These findings indicate that missing data and differences in the registries do not seem to affect the main results.

## Discussion

### Major Findings

This study found that previous stroke, advanced age (defined by cohorts aged 75-84 years and ≥85 years), hypertension, type of AF (persistent or permanent), and BMI of less than 18.5 were significant, independent risk factors associated with ischemic stroke in Japanese patients with NVAF. Notably, type of AF (persistent or permanent) and low BMI, neither of which is included in CHADS_2_ scores, were found to be novel risk factors associated with ischemic stroke. These risk factors may be useful for identifying Japanese patients with NVAF who are at risk for ischemic stroke.

### Risk Factors in CHADS_2_ Score

Individual Japanese AF registries demonstrated that advanced age (≥75 years), low body mass (BMI <18.5), previous stroke, heart failure, kidney dysfunction, anemia, and persistent or permanent AF are among the risk factors associated with ischemic stroke.^7,8,9,10,11^ Our previous pooled analysis from the J-RHYTHM Registry, Fushimi AF Registry, and Shinken Database in patients not treated with OAC (3588 patients) showed that age 75 years or older, hypertension, and previous stroke or transient ischemic attack were risk factors associated with ischemic stroke, but neither heart failure nor diabetes was.^5^ The present analysis of the larger number of patients from 5 NVAF registries (n = 12 289) supported this. Importantly, the older cohort (age ≥85 years) was associated with a higher incidence of ischemic stroke than the younger cohort (ages 75-84 years), a result consistent with findings from the Fushimi AF Registry.^17^ This fact is clinically important in societies with a high proportion of older adults such as Japan.

This study found further evidence suggesting neither heart failure nor diabetes was a risk factor associated with ischemic stroke. The Fushimi AF Registry demonstrated that although heart failure at enrollment was not significantly associated with incidence of stroke, patients admitted who experienced heart failure after enrollment, or those with a high BNP (B-type natriuretic peptide) or NT-proBNP (N-terminal pro-B-type natriuretic peptide) level, were at a risk for stroke.^18^ Furthermore, the Hokuriku-Plus AF Registry demonstrated that high BNP level was significantly associated with future thromboembolic events.^11^ These findings strongly suggest that the effect of heart failure on the incidence of stroke is dependent on the stage or severity of heart failure. Objective markers for heart failure, such as BNP/NT-proBNP, would stratify patients with NVAF diagnosed as having heart failure more accurately.

Regarding diabetes, Patti et al^19^ reported a higher risk of stroke or systemic embolism in patients with diabetes receiving insulin treatment compared with those without insulin. Notably, there was no difference in embolic events between patients with diabetes not receiving insulin and those without diabetes. These findings indicate that stratified analysis for diabetes using the pooled database will clearly be required.

The J-RHYTHM Registry showed that hypertension at enrollment is not a risk factor, but a systolic blood pressure greater than or equal to 136 mm Hg at the time closest to the event was associated with the incidence of thromboembolism.^20^ The Fushimi AF Registry showed that a systolic blood pressure greater than 150 mm Hg at enrollment was a risk factor for stroke, although history of hypertension was not.^21^ All these results indicate that blood pressure control is critical for preventing ischemic stroke in patients with NVAF. More detailed and stratified analysis using the pooled data will be of great interest.

### Novel Risk Factors for Ischemic Stroke in Japanese NVAF Patients

The Fushimi AF Registry previously found that low body weight (≤50 kg) or low BMI (<18.5) is a risk factor associated with stroke.^7,22^ A recent meta-analysis^23^ found that low BMI was associated with an increased thromboembolism risk in Asian patients with AF. More recently, an analysis of the Korean National Health Insurance Service database found that patients with AF and low body weight (<50 kg) receiving OAC had a higher incidence of ischemic stroke than those weighting 50 to 60 kg.^24^ The present study is consistent with these results, as a low BMI was a significant risk factor associated with ischemic stroke. It should be pointed out, however, that 57 patients with the single risk factor of low BMI had no ischemic stroke event during follow-up. Low BMI may not be a risk factor, but a risk modifier, such as with female sex in CHA_2_DS_2_-VASc score.^25^ Further studies are required to explore this possibility.

Persistent or permanent AF was shown to be associated with an increased risk of ischemic stroke in the Fushimi AF Registry and Hokuriku-Plus AF Registry.^8,11^ Furthermore, in the global ROCKET-AF and ENGAGE AF-TIMI 48 trials and recent meta-analysis,^26,27,28^ patients with persistent or permanent AF had more strokes or systemic embolic events than those with paroxysmal AF. The present results were consistent with those of the global studies, and when the risk for ischemic stroke is assessed, the AF type should be considered.

Lower creatinine clearance has been shown to be a risk factor of ischemic stroke in NVAF patients.^9^ In the present study, the serum creatinine level was used for the analysis and creatinine clearance was not, since the latter is determined in clinical practice by the Cockcroft-Gault equation, which includes both age and body weight, and thus is not considered as a single risk factor. In fact, when creatinine clearance was included as a variable in multivariable analysis, neither creatinine clearance nor low BMI was a significant risk factor. The present results showed that the serum creatinine level was not a significant risk factor associated with ischemic stroke in multivariable analysis. Stratified analysis in kidney dysfunction is therefore warranted.

### Limitations

This study has several limitations. First, the results were not prospectively validated by the external data. Second, 3809 patients were excluded because of the missing data. We cannot completely exclude the possibility that the lack of data may have affected the results. However, multiple imputation analysis confirmed our case analysis. Third, the follow-up period was limited to a mean of 649 days. A longer follow-up period would be preferable, but event data and follow-up periods exceeding 730 days were excluded from the present analysis to balance among the registries. Wang et al^29^ found that the ischemic stroke rate in paroxysmal and persistent patients with NVAF without OAC (mean [SD] CHADS_2_ score, 1.7 [1.4] and 2.1 [1.5], respectively) from the Chinese Atrial Fibrillation Registry study was 1.0 and 1.4 per 100 person-years in each, which were quite similar to the present results. In addition, there may be other risk factors or markers for ischemic stroke, such as BNP/NT-proBNP levels and echocardiographic values. Further studies in this area are needed.

## Conclusions

This cohort study found that previous stroke, advanced age, hypertension, type of AF, and BMI less than 18.5 were independent risk factors associated with ischemic stroke in Japanese patients with NVAF. These risk factors may be useful for identifying patients with NVAF at risk for ischemic stroke.

## Notes

Supplement. **eTable 1.** Detailed Missing Data in the Study Patients **eTable 2.** Patient Registration Period and Definition of Risk Factors in Each Registry **eTable 3.** Competing Risk Regression Model Analysis **eReferences.**

## References

[1] Kirchhof P, Benussi S, Kotecha D, 2016 ESC Guidelines for the management of atrial fibrillation developed in collaboration with EACTS. Europace. 2016;18(11):-. doi:10.1093/europace/euw295 27567465

[2] January CT, Wann LS, Calkins H, 2019 AHA/ACC/HRS Focused Update of the 2014 AHA/ACC/HRS Guideline for the Management of Patients With Atrial Fibrillation: a report of the American College of Cardiology/American Heart Association Task Force on Clinical Practice Guidelines and the Heart Rhythm Society in Collaboration With the Society of Thoracic Surgeons. Circulation. 2019;140(2):e125-e151. doi:10.1161/CIR.0000000000000665 30686041

[3] JCS Joint Working Group Guidelines for Pharmacotherapy of Atrial Fibrillation (JCS 2013). Circ J. 2014;78(8):1997-2021. doi:10.1253/circj.CJ-66-0092 24965079

[4] Okumura K, Inoue H, Atarashi H, Yamashita T, Tomita H, Origasa H; J-RHYTHM Registry Investigators Validation of CHA_2_DS_2_-VASc and HAS-BLED scores in Japanese patients with nonvalvular atrial fibrillation: an analysis of the J-RHYTHM Registry. Circ J. 2014;78(7):1593-1599. doi:10.1253/circj.CJ-14-0144 24759791

[5] Suzuki S, Yamashita T, Okumura K, Incidence of ischemic stroke in Japanese patients with atrial fibrillation not receiving anticoagulation therapy—pooled analysis of the Shinken Database, J-RHYTHM Registry, and Fushimi AF Registry. Circ J. 2015;79(2):432-438. doi:10.1253/circj.CJ-14-1131 25501800

[6] Gage BF, Waterman AD, Shannon W, Boechler M, Rich MW, Radford MJ Validation of clinical classification schemes for predicting stroke: results from the National Registry of Atrial Fibrillation. JAMA. 2001;285(22):2864-2870. doi:10.1001/jama.285.22.2864 11401607

[7] Hamatani Y, Yamashita Y, Esato M, Predictors for stroke and death in non-anticoagulated Asian patients with atrial fibrillation: the Fushimi AF Registry. PLoS One. 2015;10(11):e0142394. doi:10.1371/journal.pone.0142394 26540107 PMC4634924

[8] Takabayashi K, Hamatani Y, Yamashita Y, Incidence of stroke or systemic embolism in paroxysmal versus sustained atrial fibrillation: the Fushimi Atrial Fibrillation Registry. Stroke. 2015;46(12):3354-3361. doi:10.1161/STROKEAHA.115.010947 26514188

[9] Kodani E, Atarashi H, Inoue H, Okumura K, Yamashita T, Origasa H; J-RHYTHM Registry Investigators Impact of creatinine clearance on outcomes in patients with non-valvular atrial fibrillation: a subanalysis of the J-RHYTHM Registry. Eur Heart J Qual Care Clin Outcomes. 2018;4(1):59-68. doi:10.1093/ehjqcco/qcx032 28950373 PMC5862022

[10] Abe M, Ogawa H, Ishii M, Relation of stroke and major bleeding to creatinine clearance in patients with atrial fibrillation (from the Fushimi AF Registry). Am J Cardiol. 2017;119(8):1229-1237. doi:10.1016/j.amjcard.2017.01.005 28219663

[11] Hayashi K, Tsuda T, Nomura A, ; Hokuriku-Plus AF Registry Investigators Impact of B-type natriuretic peptide level on risk stratification of thromboembolism and death in patients with nonvalvular atrial fibrillation—the Hokuriku-Plus AF Registry. Circ J. 2018;82(5):1271-1278. doi:10.1253/circj.CJ-17-1085 29491320

[12] Tomita H, Okumura K, Inoue H, ; J-RHYTHM Registry Investigators Validation of risk scoring system excluding female sex from CHA2DS2-VASc in Japanese patients with nonvalvular atrial fibrillation—subanalysis of the J-RHYTHM Registry. Circ J. 2015;79(8):1719-1726. doi:10.1253/circj.CJ-15-0095 25971525

[13] Atarashi H, Inoue H, Okumura K, Yamashita T, Kumagai N, Origasa H; J-RHYTHM Registry Investigators Present status of anticoagulation treatment in Japanese patients with atrial fibrillation: a report from the J-RHYTHM Registry. Circ J. 2011;75(6):1328-1333. doi:10.1253/circj.CJ-10-1119 21467664

[14] Akao M, Chun YH, Wada H, ; Fushimi AF Registry Investigators Current status of clinical background of patients with atrial fibrillation in a community-based survey: the Fushimi AF Registry. J Cardiol. 2013;61(4):260-266. doi:10.1016/j.jjcc.2012.12.002 23403369

[15] Suzuki S, Yamashita T, Otsuka T, Recent mortality of Japanese patients with atrial fibrillation in an urban city of Tokyo. J Cardiol. 2011;58(2):116-123. doi:10.1016/j.jjcc.2011.06.006 21820280

[16] Ikemura N, Kohsaka S, Kimura T, Assessment of sex differences in the initial symptom burden, applied treatment strategy, and quality of life in Japanese patients with atrial fibrillation. JAMA Netw Open. 2019;2(3):e191145. doi:10.1001/jamanetworkopen.2019.1145 30924896 PMC6450322

[17] Yamashita Y, Hamatani Y, Esato M, Clinical characteristics and outcomes in extreme elderly (age >/= 85 years) Japanese patients with atrial fibrillation: the Fushimi AF Registry. Chest. 2016;149(2):401-412. doi:10.1378/chest.15-1095 26181726

[18] Iguchi M, Tezuka Y, Ogawa H, Incidence and risk factors of stroke or systemic embolism in patients with atrial fibrillation and heart failure—the Fushimi AF Registry. Circ J. 2018;82(5):1327-1335. doi:10.1253/circj.CJ-17-1155 29526914

[19] Patti G, Lucerna M, Cavallari I, Insulin-requiring versus noninsulin-requiring diabetes and thromboembolic risk in patients with atrial fibrillation: PREFER in AF. J Am Coll Cardiol. 2017;69(4):409-419. doi:10.1016/j.jacc.2016.10.069 28126158

[20] Kodani E, Atarashi H, Inoue H, ; J-RHYTHM Registry Investigators Impact of blood pressure control on thromboembolism and major hemorrhage in patients with nonvalvular atrial fibrillation: a subanalysis of the J-RHYTHM Registry. J Am Heart Assoc. 2016;5(9):e004075. doi:10.1161/JAHA.116.004075 27620886 PMC5079049

[21] Ishii M, Ogawa H, Unoki T, Relationship of hypertension and systolic blood pressure with the risk of stroke or bleeding in patients with atrial fibrillation: the Fushimi AF Registry. Am J Hypertens. 2017;30(11):1073-1082. doi:10.1093/ajh/hpx094 28575205

[22] Hamatani Y, Ogawa H, Uozumi R, Low body weight is associated with the incidence of stroke in atrial fibrillation patients—insight from the Fushimi AF Registry. Circ J. 2015;79(5):1009-1017. doi:10.1253/circj.CJ-14-1245 25740669

[23] Zhu W, Wan R, Liu F, Relation of body mass index with adverse outcomes among patients with atrial fibrillation: a meta-analysis and systematic review. J Am Heart Assoc. 2016;5(9):e004006. doi:10.1161/JAHA.116.004006 27613773 PMC5079045

[24] Lee SR, Choi EK, Park CS, Direct oral anticoagulants in patients with nonvalvular atrial fibrillation and low body weight. J Am Coll Cardiol. 2019;73(8):919-931. doi:10.1016/j.jacc.2018.11.051 30819360

[25] Nielsen PB, Skjøth F, Overvad TF, Larsen TB, Lip GYH Female sex is a risk modifier rather than a risk factor for stroke in atrial fibrillation: should we use a CHA2DS2-VA score rather than CHA2DS2-VASc? Circulation. 2018;137(8):832-840. doi:10.1161/CIRCULATIONAHA.117.029081 29459469

[26] Steinberg BA, Hellkamp AS, Lokhnygina Y, ; ROCKET-AF Steering Committee and Investigators Higher risk of death and stroke in patients with persistent vs. paroxysmal atrial fibrillation: results from the ROCKET-AF Trial. Eur Heart J. 2015;36(5):288-296. doi:10.1093/eurheartj/ehu359 25209598 PMC4313363

[27] Link MS, Giugliano RP, Ruff CT, ; ENGAGE AF-TIMI 48 Investigators Stroke and mortality risk in patients with various patterns of atrial fibrillation: results from the ENGAGE AF-TIMI 48 trial (Effective Anticoagulation with Factor Xa Next Generation in Atrial Fibrillation-Thrombolysis in Myocardial Infarction 48). Circ Arrhythm Electrophysiol. 2017;10(1):e004267. doi:10.1161/CIRCEP.116.004267 28077507

[28] Ganesan AN, Chew DP, Hartshorne T, The impact of atrial fibrillation type on the risk of thromboembolism, mortality, and bleeding: a systematic review and meta-analysis. Eur Heart J. 2016;37(20):1591-1602. doi:10.1093/eurheartj/ehw007 26888184

[29] Wang Y, Ma CS, Du X, Thromboembolic risks associated with paroxysmal and persistent atrial fibrillation in Asian patients: a report from the Chinese atrial fibrillation registry. BMC Cardiovasc Disord. 2019;19(1):283. doi:10.1186/s12872-019-1260-7 31810439 PMC6898943

