# Supplementary material for: Risk Factors Associated With Ischemic Stroke in Japanese Patients With Nonvalvular Atrial Fibrillation
Source: JAMA Netw Open. 2020 Apr 15;3(4):e202881. doi: 10.1001/jamanetworkopen.2020.2881 (PMC7160687; doi:10.1001/jamanetworkopen.2020.2881)
Supplement: Supplement. — eTable 1. Detailed Missing Data in the Study Patients eTable 2. Patient Registration Period and Definition of Risk Factors in Each Registry eTable 3. Competing Risk Regression Model Analysis eReferences. [file jamanetwopen-3-e202881-s001.pdf]

Supplementary Online Content

Okumura K, Tomita H, Nakai M, et al. Risk factors associated with ischemic stroke in Japanese patients with nonvalvular atrial fibrillation. *JAMA Netw Open*. 2020;3(4):e202881. 10.1001/jamanetworkopen.2020.2881

eTable 1. Detailed Missing Data in the Study Patients
eTable 2. Patient Registration Period and Definition of Risk Factors in Each Registry
eTable 3. Competing Risk Regression Model Analysis
eReferences.

This supplementary material has been provided by the authors to give readers additional information about their work.

© 2020 Okumura K et al. *JAMA Network Open*.

eTable 1. Detailed missing data in the study patients

	Number of missing data
Body mass index	1,824
Chronic heart failure	10
Hypertension	18
Diabetes	1
Type of atrial fibrillation	23
Creatinine	1,695
Hemoglobin	1,718
Event record during follow-up	81
Follow-up period	195
Oral anticoagulants	42

© 2020 Okumura K et al. *JAMA Network Open*.

eTable 2. Patient registration period and definition of risk factors in each registry

Registry (reference)	J-RHYTHM Registry (1-3)	Fushimi AF Registry (4)	Shinken Database (5)	Keio interhospital Cardiovascular Studies (6)	Hokuriku-Plus AF Registry (7)
Patient registration	From January, 2009 to July, 2009	From February, 2011 ~ ongoing	From June, 2004 ~ ongoing	From January, 2012 to March, 2018	From May, 2013 to May, 2014
"C" Congestive heart failure	● Recent heart failure based on the criteria of $CHADS_2$ score<br>● Clinically diagnosed heart failure	● History of hospitalization for heart failure<br>● Symptoms of heart failure (NYHA≥2)<br>● Left ventricular ejection fraction <40%	● Symptoms of heart failure (NYHA≥2)	● History of hospitalization for heart failure<br>● Symptoms of heart failure	● History of hospitalization for heart failure<br>● Symptoms of heart failure<br>● Treatment for heart failure
"H" Hypertension	● Blood pressure ≥140/90 mmHg<br>● Antihypertensive drug<br>● History of hypertension	● Blood pressure >140/90 mmHg<br>● Antihypertensive drug	● Blood pressure >140/90 mmHg<br>● Antihypertensive drug	● Blood pressure ≥140/90 mmHg<br>● Antihypertensive drug<br>● History of hypertension	● Blood pressure >140/90 mmHg<br>● Antihypertensive drug
"D" Diabetes	● HbA1c ≥6.5%<br>● Treatment for diabetes	● HbA1c >6.5%<br>● Treatment for diabetes	● HbA1c >6.5%<br>● Treatment for diabetes	● HbA1c >6.5%<br>● Fasting plasma glucose >126 mg/dL<br>● Random plasma glucose >200 mg/dL<br>● Treatment for diabetes	● HbA1c >6.5%<br>● Fasting plasma glucose >126 mg/dL<br>● Random plasma glucose >200 mg/dL<br>● Treatment for diabetes
"V" Vascular diseases	● Coronary artery disease	● Myocardial infarction<br>● Peripheral artery disease	● Myocardial infarction<br>● Peripheral artery disease	● Coronary artery disease<br>● Peripheral artery disease<br>● Large vessel disease	● Coronary artery disease<br>● Peripheral artery disease<br>● Large vessel disease

NYHA indicates New York Heart Association Functional Classification, HbA1c; Hemoglobin A1c.

© 2020 Okumura K et al. *JAMA Network Open*.

eTable 3. Competing risk regression model analysis

	Univariate		Multivariable		Stepwise	
	SHR (95%CI)	p-value	SHR (95%CI)	p-value	SHR (95%CI)	p-value
Age (<75 years)	Reference		Reference		Reference	
75-84 years	1.63 (1.26-2.11)	<0.001	1.60 (1.19-2.14)	0.002	1.74 (1.31-2.31)	<0.001
≥85 years	2.67 (1.89-3.78)	<0.001	2.04 (1.33-3.12)	0.001	2.41 (1.59-3.64)	<0.001
Female	1.36 (1.05-1.76)	0.02	1.20 (0.90-1.59)	0.22		
CHF	1.48 (1.14-1.93)	0.004	1.08 (0.81-1.44)	0.59		
Hypertension	1.63 (1.17-2.27)	0.004	1.51 (1.07-2.13)	0.02	1.60 (1.14-2.24)	0.007
Diabetes	1.21 (0.90-1.62)	0.21	1.09 (0.80-1.48)	0.58		
Previous stroke	3.08 (2.36-4.04)	<0.001	2.72 (2.04-3.62)	<0.001	2.75 (2.07-3.65)	<0.001
Vascular disease	1.34 (0.96-1.87)	0.09	1.03 (0.73-1.46)	0.86		
Type of AF (persistent/permanent)	1.61 (1.23-2.11)	<0.001	1.58 (1.18-2.11)	0.002	1.59 (1.21-2.10)	0.001
Low BMI <18.5 kg/m^2	1.95 (1.33-2.86)	0.001	1.46 (0.98-2.19)	0.07	1.54 (1.04-2.29)	0.03
Creatinine ≥1.0 mg/dL	1.47 (1.13-1.90)	0.004	1.21 (0.92-1.60)	0.17		
Low hemoglobin	1.72 (1.33-2.24)	<0.001	1.18 (0.88-1.58)	0.28		
No oral anticoagulant	1.49 (1.14-1.95)	0.004	1.86 (1.38-2.51)	<0.001	1.86 (1.38-2.50)	<0.001

SHR indicates subhazard ratio, CI; confidence interval, CHF; congestive heart failure, AF; atrial fibrillation, BMI; body mass index.

© 2020 Okumura K et al. *JAMA Network Open*.

eReferences

1. Atarashi H, Inoue H, Okumura K, Yamashita T, Origasa H. Investigation of optimal anticoagulation strategy for stroke prevention in Japanese patients with atrial fibrillation-the J-RHYTHM Registry study design. J Cardiol. 2011;57(1):95-99.

2. Kodani E, Atarashi H, Inoue H, et al. Impact of blood pressure control on thromboembolism and major hemorrhage in patients with non-valvular atrial fibrillation: A subanalysis of the J-RHYTHM Registry. J Am Heart Assoc. 2016;5(9):e004075.

3. Okumura K, Inoue H, Atarashi H, Yamashita T, Tomita H, Origasa H. Validation of CHA_2DS_2-VASc and HAS-BLED scores in Japanese patients with nonvalvular atrial fibrillation: an analysis of the J-RHYTHM Registry. Circ J. 2014;78(7):1593-1599.

4. Akao M, Chun YH, Wada H, et al. Current status of clinical background of patients with atrial fibrillation in a community-based survey: the Fushimi AF Registry. J Cardiol. 2013;61(4):260-266.

5. Suzuki S, Otsuka T, Sagara K, et al. Nine-year trend of anticoagulation use, thromboembolic events, and major bleeding in patients with non-valvular atrial fibrillation- Shinken Database Analysis. Circ J. 2016;80(3):639-649.

6. Ikemura N, Kohsaka S, Kimura T, et al. Assessment of sex differences in the initial symptom burden, applied treatment strategy, and quality of life in Japanese patients with atrial fibrillation. JAMA Netw Open. 2019;2(3):e191145.

7. Hayashi K, Tsuda T, Nomura A, et al. Impact of B-type natriuretic peptide level on risk stratification of thromboembolism and death in patients with nonvalvular atrial fibrillation- The Hokuriku-Plus AF Registry. Circ J. 2018;82(5):1271-1278.

© 2020 Okumura K et al. *JAMA Network Open*.
